# A PAF receptor antagonist inhibits acute airway inflammation and late-phase responses but not chronic airway inflammation and hyperresponsiveness in a primate model of asthma

**DOI:** 10.1155/S0962935192000577

**Published:** 1992

**Authors:** R. H. Gundel, C. D. Wegner, H. O. Heuer, L. G. Letts

**Affiliations:** Department of Pharmacology Boehringer Ingelheim Pharmaceuticals Inc. 900 Ridgebury Road Ridgefield CT 06877 USA

## Abstract

We have examined the effects of a PAF receptor antagonist, WEB 2170, on several indices of acute and chronic airway inflammation and associated changes in lung function in a primate model of allergic asthma. A single oral administration WEB 2170 provided dose related inhibition of the release of leukotriene C_4_ (LTC_4_) and prostaglandin D_2_ (PGD_2_) recovered and quantified in bronchoalveolar lavage (BAL) fluid obtained during the acute phase response to inhaled antigen. In addition, oral WEB 2170 treatment in dual responder primates blocked the acute influx of neutrophils into the airways as well as the associated late-phase airway obstruction occurring 6 h after antigen inhalation. In contrast, a multiple dosing regime with WEB 2170 (once a day for 7 consecutive days) failed to reduce the chronic airway inflammation (eosinophilic) and associated airway hyperresponsiveness to inhaled methacholine that is characteristic of dual responder monkeys. Thus, we conclude that the generation of PAF following antigen inhalation contributes to the development of lipid mediators, acute airway inflammation and associated late-phase airway obstruction in dual responder primates; however, PAF does not play a significant role in the maintenance of chronic airway inflammation and associated airway hyperresponsiveness in this primate model.


Mediators of Inflammation 1, 379–384 (1992)

**We have examined the effects of a PAF receptor antagonist, WEB 2170, on several indices of acute and chronic airway inflammation and associated changes in lung function in a primate model of allergic asthma. A single oral administration WEB 2170 provided dose related inhibition of the release of leukotriene $C_4$ ($LTC_4$) and prostaglandin $D_2$ ($PGD_2$) recovered and quantified in bronchoalveolar lavage (BAL) fluid obtained during the acute phase response to inhaled antigen. In addition, oral WEB 2170 treatment in dual responder primates blocked the acute influx of neutrophils into the airways as well as the associated late-phase airway obstruction occurring 6 h after antigen inhalation. In contrast, a multiple dosing regime with WEB 2170 (once a day for 7 consecutive days) failed to reduce the chronic airway inflammation (eosinophilic) and associated airway hyperresponsiveness to inhaled methacholine that is characteristic of dual responder monkeys. Thus, we conclude that the generation of PAF following antigen inhalation contributes to the development of lipid mediators, acute airway inflammation and associated late-phase airway obstruction in dual responder primates; however, PAF does not play a significant role in the maintenance of chronic airway inflammation and associated airway hyperresponsiveness in this primate model.**

**Key words:** Acute and chronic airway inflammation, Airway hyperresponsiveness, Late phase response, Lipid mediators, PAF

### A PAF receptor antagonist inhibits acute airway inflammation and late-phase responses but not chronic airway inflammation and hyperresponsiveness in a primate model of asthma

**R. H. Gundel,[CA] C. D. Wegner, H. O. Heuer and L. G. Letts**

Department of Pharmacology, Boehringer Ingelheim Pharmaceuticals, Inc., 900 Ridgebury Road, Ridgefield, CT 06877, USA

[CA] Corresponding Author

### Introduction

Platelet activating factor (PAF) is a naturally occurring phospholipid with potent biological actions that mimic the pathophysiology of asthma.[1] In the lung PAF mediates a wide range of effects including bronchoconstriction,[2] mucus hypersecretion,[3] increases in vascular permeability,[4] chemotaxis of pro-inflammatory cells[5] and increases in bronchial responsiveness.[6] In addition to its direct effects in the lung, PAF may also act indirectly by inducing arachidonic acid metabolism resulting in the formation of other lipid mediators that also have potent bronchoconstrictor and pro-inflammatory effects in the lungs.[7] Recent studies suggest that PAF binds to specific receptors located on the surface of a variety of cells and mediates its biological effects via activation of a G protein, phosphoinositide metabolism and subsequent increases in intracellular calcium levels.[8] The presence of PAF receptors lends itself to pharmacological intervention with selective receptor antagonists.

The hetrazepine WEB 2170 (bepafant) is a recently synthesized and characterized selective PAF receptor antagonist that has potent activity inhibiting PAF induced responses in a variety of *in vitro* and *in vivo* systems.[9] As such, WEB 2170 is a potential therapeutic agent that may be useful in the treatment of asthma and other related inflammatory diseases. We have previously reported that late-phase responder monkeys have a persistent, baseline airway eosinophilia and airway hyperresponsiveness prior to antigen inhalation.[10] Following inhalation challenge, these monkeys have an immediate (acute) and late-phase airway response that is associated with a large, transient influx of neutrophils into the airways superimposed on the baseline chronic airway eosinophilia. Thus, this model enables one to examine the effects of treatment on both acute and chronic airway inflammation and respective changes in airway function.

The purpose of this study was to examine the effects of WEB 2170 on antigen induced acute airway inflammation and associated changes in airway function. In addition, we also examined its efficacy in reducing chronic airway inflammation and airway hyperresponsiveness, characteristic of dual responder monkeys.

### Methods

*Animals:* Animals used in this study were wild caught, adult male cynomolgus monkeys (*Macaca*

© 1992 Rapid Communications of Oxford Ltd

*fascicularis*) weighing 3.5–7.5 kg and demonstrating a naturally occurring respiratory hypersensitivity to *Ascaris suum* extract. Monkeys were housed individually in specially designed open mesh cages, provided food twice a day and water *ad libitum*. All protocols used in this study were approved by the Animal Care and Use Committee at Boehringer Ingelheim Pharmaceuticals, Inc., Ridgefield, CT.

*General protocol:* For each study, monkeys were anaesthetized with an intramuscular injection of ketamine hydrochloride (4 mg/kg) and xylazine (1 mg/kg), intubated with a cuffed endotracheal tube (5.5 internal diameter) and seated in an upright position in a specially designed support chair. Ketamine (4 mg/kg, i.m.) was used to supplement anaesthesia as needed.

*Acute airway inflammation and late-phase responses:* Test compound or vehicle were administered by gavage approximately 1 h before antigen challenge. Baseline respiratory system resistance (Rrs) was monitored over a 15 min time period after which antigen inhalation challenge was administered. Following antigen challenge Rrs was monitored for 10 min (peak immediate response) after which the animal was removed from the chair, placed in the supine position and bronchoalveolar lavage (BAL) was performed with a pediatric bronchoscope. The measurement of lipid mediators in BAL fluid recovered after antigen inhalation was determined as previously described.[11] Following BAL, each animal was allowed to recover from anaesthesia and returned to their cage. To monitor the late-phase response, each animal was re-anaesthetized with the ketamine/xylazine mixture, intubated, seated in the support chair and Rrs was monitored over a 15 min time period. Rrs measurements were recorded at 4, 6, 8, 10 and 24 h post antigen inhalation. In order to examine antigen induced changes in airway cellular composition and BAL mediators, BAL was performed 6 h after antigen challenge on the opposite lung that was lavaged during the immediate response. The results of drug treatment studies were compared to bracketing control studies in each animal. Experiments were separated by a minimum of 2 weeks. Animals were fasted for approximately 18 h prior to use.

*Chronic airway inflammation and airway hyperresponsiveness:* In a separate series of studies, the effects of a multiple dosing regime of WEB 2170 on chronic airway inflammation and hyperresponsiveness was examined. On study day 0, animals were anaesthetized (ketamine/xylazine mixture) and airway responsiveness was evaluated by performing an inhaled methacholine concentration response determination. Following this procedure, airway cellular composition was assessed by BAL. On study day 1, each animal received either vehicle or WEB 2170 (10 mg/kg, p.o.) each day for 7 consecutive days (cross over study design). On study day 7, airway responsiveness to inhaled methacholine was evaluated and airway cellular composition was assessed by performing BAL on the opposite lung lavaged on day 0 of the study.

*Aerosol delivery system and inhalation challange:* Aerosol inhalation challenges were administered by compressed air nebulization and intermittent positive pressure breathing with a Bird Mark 7A respirator and micronebulizer (Bird Corporation, model 8158). For antigen inhalation, each challenge consisted of 15 breaths per min (maximum inspiratory pressure of 20 cm $H_2O$) for 2 min. *Ascaris suum* extract was diluted with phosphate buffered saline (PBS, pH 7.4) to the appropriate concentration for each animal (dose required to cause a 200–300% increase in Rrs). Antigen challenges were separated by a minimum of 14 days for each animal. For inhaled methacholine challenges, each challenge consisted of 15 breaths per min for 1 min. A vehicle (phosphate buffered saline) inhalation was performed prior to the first dose of methacholine.

*Measurement of respiratory system resistance (Rrs):* Respiratory system impedance (Zrs) was measured by discrete frequency (4–40 Hz in eleven equal logarithmic steps) sinusoidal forced oscillations superimposed on tidal breathing as described by Wegner and co-workers.[12] The mean of the real or in-phase component of Zrs over the entire frequency range was then computed to provide a single value representation of Rrs.

*Bronchoalveolar lavage (BAL):* BAL was performed by placing each monkey in the supine position and guiding a fibre-optic bronchoscope past the carina and wedging into a fifth to seventh generation bronchi. A 15 ml aliquot of bicarbonate buffered saline (pH 7.4, 23°C) was infused and gently aspirated through a channel in the bronchoscope.

*Concentration of BAL fluid and measurement of lipid mediators:* Collected BAL fluid samples were concentrated and lipid mediator content analysed as previously described.[11] Briefly, collected samples were centrifuged at 600 × *g* for 10 min at 4°C to remove cells and particulates, after which the supernatants were diluted with cold ethanol (1:5 dilution). BAL supernatants were again centrifuged at 600 × *g* for 10 min to remove residual precipitate. Samples were then concentrated approximately five-fold under vacuum after which the samples could be stored at −70°C until analysis by high pressure liquid chromatography (HPLC) and radioimmunoassay (RIA).

*Histochemistry:* BAL cells were evaluated with the use of cytocentrifuge preparations stained with Diff-Quick stain. Differential cell counts were determined by counting 200 cells, and the percentage of each cell type was recorded. Total BAL cells were determined with the use of a Coulter Counter.

*Statistical analysis:* Statistical analysis of the level of lipid mediators, cell influx into the lungs and immediate and late-phase responses was performed using a two-way analysis of variance and Duncan's multiple comparison $t$-test where a $p$ value less than 0.05 was considered statistically significant. The percent inhibition of mediator release, acute and late-phase responses were calculated using the mean value of the two bracketing control studies performed for each dose of WEB 2170 tested.

### Results

*Antigen induced lipid mediator release:* The effects of antigen inhalation during control (vehicle) and WEB 2170 treatment studies on the level of BAL $LTC_4$ and $PGD_2$ are shown in Table 1. Prior to antigen inhalation $LTC_4$ and $PGD_2$ were present in concentrated BAL fluid but at very low levels close to the detectable limits of the RIA. Antigen challenge resulted in a significant increase in BAL $LTC_4$ and $PGD_2$ recovered in BAL fluid 20 min post challenge. Pretreatment with WEB 2170 provided a dose related inhibition of antigen induced increases in BAL $LTC_4$ and $PGD_2$.

*Acute airway inflammation and late-phase obstruction:* Antigen inhalation caused an acute bronchoconstriction, usually peaking 5 to 10 min post-challenge and a late-phase response that peaked 6 to 8 h post-challenge and resolved by 24 h. WEB 2170 treatment did not significantly reduce the peak acute bronchoconstriction (0–10 min) but did inhibit the late-phase response in a dose related manner (Fig. 1).

Airway cellular composition before and after antigen inhalation is shown in Table 2. Prior to antigen inhalation, there were a large numbers of

Table 1. Mediators recovered in monkey BAL fluid

| Challenge | i-$LTC_4$ (ng) | i-$PGD_2$ (ng) |
|---|---|---|
| PBS | 2 ± 1 | 2 ± 1 |
| *Ascaris* | 45 ± 9 | 32 ± 7 |
| + WEB 2170 (0.3 mg/kg) | 41 ± 6 | 28 ± 16 |
| (1 mg/kg) | 32 ± 12 | 16 ± 13 |
| (3 mg/kg) | 21 ± 8* | 16 ± 8* |
| (10 mg/kg) | 17 ± 10* | 15 ± 9* |
| (30 mg/kg) | 17 ± 8* | 13 ± 6* |

Levels of i-$LTC_4$ and i-$PGD_2$ expressed as the total amount recovered in BAL fluid. *$p \leq 0.05$. Data are the mean ± SEM.

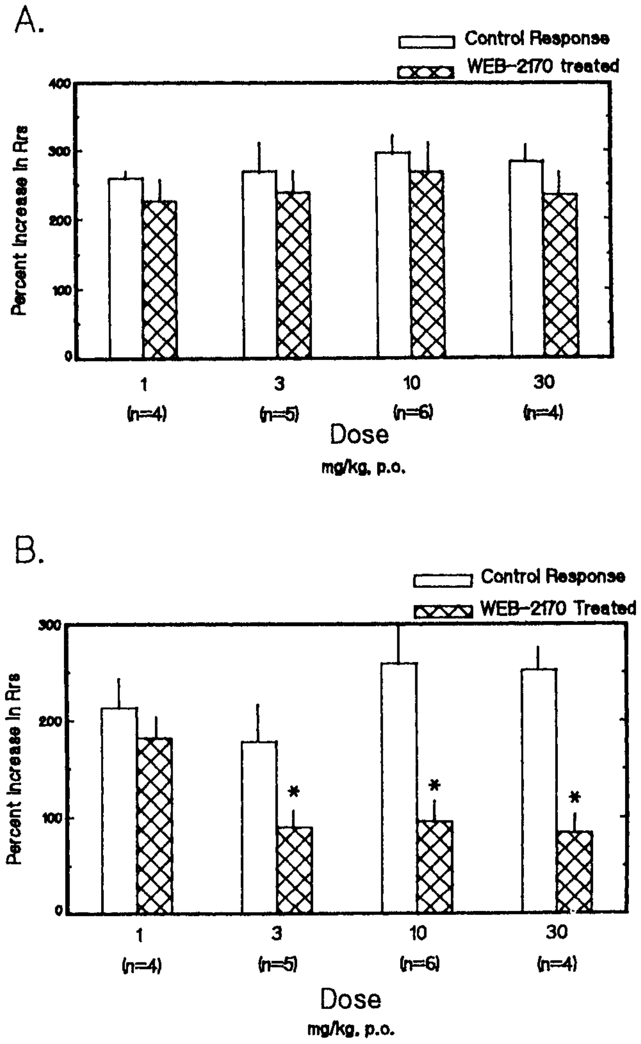

FIG. 1. (A) Effects of WEB 2170 treatment on the peak percent increase in Rrs during the immediate response to inhaled antigen. WEB 2170 (1 h prior to challenge) had no effect on the immediate response. (B) Effects of WEB 2170 treatment on the peak percentage increase in Rrs during the late-phase response. WEB 2170 significantly reduced the late-phase response at 3, 10 and 30 mg/kg doses. *Indicates statistical significance ($p \leq 0.05$).

eosinophils and very small numbers of neutrophils recovered by BAL. Antigen inhalation resulted in a large influx of neutrophils into the airways that was temporally associated (6 h) with the occurrence of the late-phase airway obstruction. WEB 2170 at doses of 10 and 30 mg/kg significantly inhibited the late-phase associated influx of neutrophils into the airways (Table 3).

*Chronic airway inflammation and airway hyperresponsiveness:* The effect of multiple doses of WEB 2170 on the baseline airway eosinophilia is shown in Fig. 2A. WEB 2170 treatment did not significantly reduce the number of eosinophils recovered by BAL on day 7 compared to day 0 of the study. Similarly, WEB 2170 treatment had no effect on the airway hyperresponsiveness to inhaled methacholine (Fig. 2B).

**Table 2.** Airway cellular composition

| Time | % Macro | % Lymph | % Neut | % Eos | Total cells ( $\times 10^5$/ml) |
|---|---|---|---|---|---|
| Before antigen | 39 ± 9<br>(178) | 5 ± 1<br>(22.8) | 8 ± 3<br>(36.5) | 48 ± 6<br>(219) | 4.56 ± 0.63 |
| 6 h post-antigen | 22 ± 7<br>(175) | 4 ± 2<br>(31.7) | 59 ± 8*<br>(468)* | 15 ± 5<br>(119) | 7.93 ± 0.89* |

*$p \leq 0.05$.
Figures in parentheses are the number of cells $\times$ $10^3$/ml BAL. Data are the mean ± SEM, $n = 12$.

**Table 3.** Effects of WEB 2170 on neutrophil influx

| Compound | Dose (mg/kg) | *n* | Neutrophil influx ( $\times 10^3$/ml) | | |
|---|---|---|---|---|---|
| | | | Control[1] | Drug treatment | % Inhibition |
| WEB 2170 | 1.0 | 4 | 235 ± 27 | 303 ± 69 | 0 |
| | 3.0 | 5 | 354 ± 38 | 192 ± 52 | 45 |
| | 10.0 | 6 | 323 ± 48 | 95 ± 61 | 71 |
| | 30.0 | 4 | 390 ± 68 | 129 ± 36 | 67 |

Data are mean ± SEM. [1] Control values represent data from bracketing control studies.
*$p \leq 0.05$.

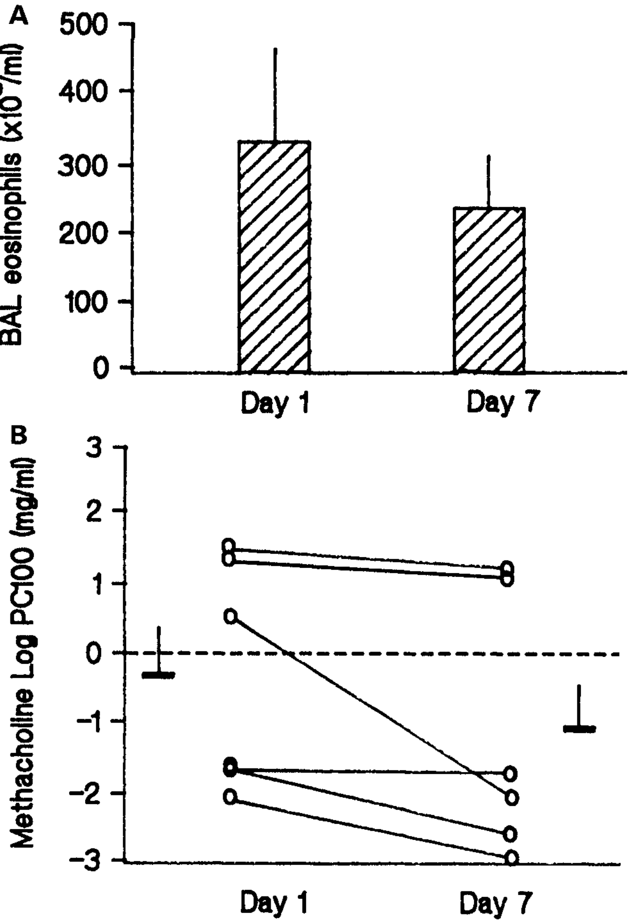

FIG. 2. (A) Effects of WEB 2170 treatment on the number of eosinophils recovered by BAL. Multiple dosing (10 mg/kg/day, p.o.) of WEB 2170 had no effect on the number of resident eosinophils in the airways of dual responder primates. Data are the mean ± SEM, $n = 6$. (B) Airway responsiveness to inhaled methacholine prior to multiple dosing with WEB 2170 (day 1) and after (day 7). WEB 2170 treatment showed no improvement in airway hyperresponsiveness to inhaled methacholine. Individual data for each animal are shown as well as the mean ± SEM for the group.

### Discussion

PAF is a pro-inflammatory mediator that has potent effects in the airways that mimic the pathophysiology of asthma. Recent studies demonstrate that PAF mediates it potent biological effects by binding to specific receptors on the cell surface.[13] WEB 2170 is a potent PAF receptor antagonist that blocks the effects of PAF *in vitro* and *in vivo*.[9] In the current study, we have examined the effects of WEB 2170 in a primate model of acute and chronic airway inflammation and associated changes in lung function. The results of this study demonstrate that WEB 2170 inhibits the production and release of arachidonic acid metabolites in the lung during the immediate response to inhaled antigen (0–10 min) as well as the late-phase airway obstruction. WEB 2170 also significantly inhibited the acute neutrophilic influx into the lungs that is associated with late-phase airway obstruction. In contrast, WEB 2170 had no effect on the baseline airway eosinophilia nor did it reduce the airway hyperresponsiveness to inhaled methacholine characteristic of dual responder monkeys.

Previous studies have demonstrated that PAF stimulates the generation of leukotrienes from human neutrophils at concentrations as low as $10^{-10}$ M.[8] Furthermore, PAF stimulates leukotriene synthesis *in vivo* in several species including the cat and rat.[14,15] Our study demonstrates that a PAF receptor antagonist inhibits the production and release of arachidonic acid metabolites ($LTC_4$ and $PGD_2$) in the lung following antigen challenge. Although we significantly reduced the production of arachidonic acid metabolites, we did not see a reduction in the peak increase in Rrs during the immediate response to inhaled antigen. This observation is consistent with the results of a previous study in which we demonstrated that lipid mediators contribute to the duration of the immediate response and not to the peak bronchoconstriction.[11] In the present study we did not monitor the duration of the immediate response to antigen but focused on the measurement of lipid mediators in BAL fluid. While the cellular sources of PAF, $LTC_4$ and $PGD_2$ were not addressed in this study, other studies have demonstrated that activated eosinophils generate large amounts of PAF and leukotrienes.[16,17] We have previously reported that late-phase responder monkeys have higher numbers of eosinophils prior to antigen challenge compared to single responder monkeys.[10] The late-phase responder monkeys utilized in this study had high levels of eosinophils (approximately 50% of the airway cells) prior to antigen challenge. Thus, the eosinophil may be an important source of potent bronchoactive arachidonic acid metabolites that may contribute to the occurrence of a late-phase response following antigen inhalation. Other cells, including mast cells and macrophages, may also contribute to lipid mediator production in the lung and may also be affected by WEB 2170.

In sensitized guinea-pigs WEB 2170 in combination with an antihistamine blocks antigen induced acute airway constriction suggesting that the synthesis of PAF contributes to airway narrowing.[18] Other studies have suggested that PAF may mediate changes in pulmonary function by causing the synthesis of other bronchoactive compounds. For example, Abraham and co-workers have demonstrated that WEB 2086 (an analogue of WEB 2170) inhibits antigen induced late-phase bronchoconstriction responses in sheep.[19] PAF induced bronchoconstriction was blocked by WEB 2086 and by a leukotriene receptor antagonist while indomethacin and chlorpheniramine had no effect. Furthermore, aerosolized $LTD_4$ induced bronchoconstriction was not blocked by WEB 2086, thus suggesting that PAF induced bronchoconstriction occurs via the generation of leukotrienes. In this study, it was hypothesized that WEB 2086 blocked the late-phase response by inhibiting the production and release of leukotrienes since leukotriene receptor antagonists and leukotriene synthesis inhibitors show similar efficacy.[20] Our study extends these observations and provides direct evidence of WEB 2170 mediated leukotriene synthesis inhibition immediately following antigen challenge as well as the dose dependent inhibition of the late-phase airway obstruction. Our results with WEB 2170 are similar to what we have observed with a selective 5–lipoxygenase inhibitor.[21] Thus, our results with WEB 2170 and a leukotriene synthesis inhibitor are consistent with the observations of Abraham and associates.[19]

In the primate, the late-phase obstruction response is chronologically associated with a transient influx of neutrophils into the airways. We have recently demonstrated with the use of monoclonal antibodies against cellular adhesion proteins that inhibiting the influx of neutrophils after antigen challenge will block the occurrence of the late-phase obstruction.[22] These data suggest that the recruitment and infiltration of neutrophils into the lungs is necessary for the development of the late-phase response. Attempts to consistently measure lipid mediators in BAL fluid during the late-phase response have been unsuccessful. This could reflect the limitations of our assay methods (BAL) or may suggest the involvement of other mechanisms and/or mediators in the late-phase airway obstruction. In the present study, WEB 2170 inhibited the influx of neutrophils into the airways and significantly reduced the late-phase response. The mechanism by which WEB 2170 inhibits cell influx and late-phase responses is not known. PAF is a potent chemotactic agent for neutrophils and therefore the direct antagonism of the PAF receptor by WEB 2170 may be involved. In addition, leukotriene $B_4$ ($LTB_4$) is a potent chemotactic agent for neutrophils and may also be involved in cell recruitment into the lungs after antigen challenge. While in our study we have not measured $LTB_4$ directly, it may be that in addition to inhibiting $LTC_4$ and $PGD_2$ production and release during the immediate response, WEB 2170 may be inhibiting $LTB_4$ production and thereby inhibiting neutrophil influx. Similar results on antigen induced cellular infiltrates were reported by Soler and colleagues[23] in studies with allergic sheep. In these studies, pretreatment with WEB 2086 (before antigen challenge) significantly reduced the influx of neutrophils into the airways after antigen challenge, suggesting that PAF is generated during the immediate response and mediating the development of airway inflammation.

Our study is the first to demonstrate that a PAF receptor antagonist does not reduce chronic airway inflammation and airway hyperresponsiveness. A 7 day dosing regime of WEB 2170 had no effect on the chronic airway eosinophilia and hyperresponsiveness to inhaled methacholine in dual responder monkeys. Several possibilities exist as to why we observed no effect on chronic airway inflammation and hyperresponsiveness. It is possible that a 7 day dosing treatment is not a sufficient enough time to reduce the chronic airway inflammation present in dual responder monkeys. However, recently we demonstrated that a 7 day dosing regime with dexamethasone (0.2 mg/kg, i.m.) significantly reduced the chronic airway inflammation and airway hyperresponsiveness which would argue against this possibility.[24] Secondly, it may be that the dose of WEB 2170 and the dosing regimen selected for this study was inappropriate. The dose was selected based on efficacy in antigen induced mediator release and inhibition of the late-phase response. It may be that far higher doses and more frequent dosing (i.e., b.i.d.) are needed to significantly reduce chronic airway inflammation. On the other hand, it could be that these high doses may result in an up-regulation of PAF receptors. Since the duration of WEB 2170 is approximately 10–12 h after oral dosing, it is possible that our lack of efficacy in reducing chronic inflammation is a reflection of a rebound phenomenon. This could result from the kinetics of receptors being occupied, resulting in up-regulation occurring at a time when no circulating levels of WEB 2170 were present.

Our findings could have profound implications to the clinical efficacy of PAF receptor antagonists in asthma, a disease with a recently recognized important chronic inflammatory component. In fact, the existing clinical data so far does not support a role for PAF in chronic asthma. Several studies have failed to show inhibition of antigen induced acute and late-phase reactions with PAF antagonists.[25–27] In addition, results from a recent phase II steroid sparing study in chronic asthma indicate that WEB 2086 has no steroid sparing effects (S. T. Holgate, personal communication).

In summary, we have demonstrated that pretreatment with a specific PAF receptor antagonist, WEB 2170, effectively reduces acute airway inflammation and associated changes in lung function. WEB 2170 treatment inhibited the release of arachidonic acid metabolites in the lung produced during the immediate response to inhaled antigen. Furthermore, WEB 2170 blocked the acute infiltration of neutrophils into the airways and the occurrence of a late-phase airway obstruction. However, WEB 2170 failed to reduce the chronic baseline airway eosinophilia and airway hyperresponsiveness characteristic of dual responder monkeys. These results raise serious questions to the possibility of PAF receptor antagonists as a novel therapeutic approach to the treatment of bronchial asthma.

### References

1. Barnes PJ, Chung KF, Page CP. Platelet activating factor as a mediator of allergic disease. *J Allergy Clin Immunol* 1988; **81**: 919–934.
2. Rubin AE, Smith LJ, Patterson R. The bronchoconstricting properties of platelet activating factor in humans. *Am Rev Respir Dis* 1987; **136**: 1145–1151.
3. Wirtz H, Lang M, Sannwald U, Hahn H. Mechanism of platelet activating factor (PAF)-induced secretion of mucus from tracheal submucosal glands in ferret. *Fed Proc* 1986; **45**: 418–421.
4. Bjork J, Smedegard G. Acute microvascular effects of PAF-acether, as studied by intravital microscopy. *Eur J Pharmacol* 1983; **96**: 87–94.
5. Wardlaw AJ, Moqbel R, Cromwell O, Kay AB. Platelet activating factor: a potent chemotactic and chemokinetic factor for human eosinophils. *J Clin Invest* 1986; **78**: 1701–1706.
6. Cuss FM, Dixon CMS, Barnes PJ. Effects of inhaled platelet activating factor on pulmonary function and bronchial responsiveness in man. *Lancet* 1986; **ii**: 189–192.
7. Chilton FH, O'Flaherty JT, Walsh CE, *et al.* Platelet activating factor stimulation of the lipoxygenase pathway in polymorphonuclear leukocytes stimulated by 1–O–alkyl–2–O–acetyl–sn–glycero–3–phosphocholine. *J Biol Chem* 1982; **257**: 5402–5409.
8. McColl SR, Krump E, McDonald PP, Braquet M, Naccache PH, Borgeat P. Enhancement of platelet activating factor induced leukotriene synthesis in neutrophils by granulocyte-macrophage colony stimulating factor (GM-CSF): Studies in the mechanism of action of GM-CSF. *J Lipid Med* 1990; **2**: 5119–5127.
9. Heuer HO, Casal-Stanzel J, Muacevic G, Weber KH. Pharmacological activity of Bepafant (WEB-2170), a new and selective hetrazepinoic antagonist of platelet activating factor. *J Pharm Exp Ther* 1991; **255**: 962–968.
10. Gundel RH, Wegner CD, Letts LG. Antigen-induced acute and late-phase responses in primates. *Am Rev Respir Dis* 1992; **146**: 369–373.
11. Gundel RH, Kinkade P, Torcellini CA, *et al.* Antigen-induced mediator release in primates. *Am Rev Respir Dis* 1991; **144**: 76–82.
12. Wegner CD, Jackson AC, Berry JD, Gillespie JR. Respiratory mechanics in monkeys measured by forced oscillations. *Ann Rev Physiol* 1984; **55**: 47–61.
13. Naccache PH, Molski TFP, Volpi M, Becker EL, Sha'afi RI. Unique inhibitory profile of platelet activating factor induced calcium mobilization, polyphophoinsositide turnover and granule enzyme secretion in rabbit neutrophils towards pertussin toxin and phorbol esters. *Biochem Biophys Res Commun* 1985; **130**: 677–682.
14. Lefer AM, Roth DM, Lefer DJ, Smith JB. Potentiation of leukotriene formation in pulmonary and vascular tissue. *Arch Pharmacol* 1984; **326**: 186–189.
15. Voelkel NF, Warthen S, Reeves JT, Henson PM, Murphy RC. Nonimmunological production of leukotrienes induced by platelet activating factor. *Science* 1982; **218**: 286–288.
16. Kauffman HF, Van der Belt B, de Monchy JGR, Bolens H, Koeter GH, de Vries K. Leukotriene C4 production by normal-density and low density eosinophils of atopic individuals and other patients with eosinophilia. *J Allergy Clin Immunol* 1987; **79**: 611–619.
17. Lee TC, Lenihan DJ, Malone B, Roddy LL, Wasserman SI. Increased biosynthesis of platelet activating factor in activated human eosinophils. *J Biol Chem* 1984; **259**: 5526–5530.
18. Heuer HO. Inhibition of active anaphylaxis in mice and guinea-pigs by the new hetrazepinoic PAF antagonist bepafant (WEB 2170). *Eur J Pharmacol* 1991; **199**: 157–163.
19. Abraham WM, Stevenson JS, Garrido R. A possible role for platelet activating factor in allergen-induced late-responses. Modification by a selective antagonist. *J Appl Physiol* 1989; **66**: 2351–2357.
20. Abraham WM. The role of leukotrienes in allergen-induced late responses in allergic sheep. *Ann NY Acad Sci* 1988; **524**: 260–270.
21. Gundel RH, Torcellini CA, Clarke CC, Desai S, Lazer ES, Wegner CD. The effects of a 5-lipoxygenase inhibitor on antigen-induced mediator release, late-phase bronchoconstriction and cellular infiltrates in primates. *Adv Leuk Prost Res* 1991; **21**: 457–460.
22. Gundel RH, Wegner CD, Torcellini CA, *et al.* Role of ELAM-1 and ICAM-1 in antigen-induced acute airway inflammation and late-phase bronchoconstriction. *Am Rev Respir Dis* 1991; **143**: A42.
23. Soler M, Sielczak MW, Abraham WM. A PAF-antagonist blocks antigen-induced airway hyperresponsiveness and inflammation in sheep. *J Appl Physiol* 1989; **67**: 406–413.
24. Gundel RH, Wegner CD, Torcellini CA, Letts LG. The role of intercellular adhesion molecule-1 in chronic airway inflammation. *Clin Exp Allergy* 1992; **22**: 569–575.
25. Freitag A, Watson RM, *et al.* The effects of treatment with an oral platelet activating factor antagonist (WEB 2086) on allergen induced asthmatic responses in human subjects. *Am Rev Respir Dis* 1991; **143**: A157.
26. Wilkens H, Wilkens JH, *et al.* Effects of inhaled PAF-antagonist (WEB 2086) on allergen-induced early and late asthmatic responses and increased bronchial responsiveness to methacholine. *Am Rev Respir Dis* 1991; **143**: A812.
27. Bel EH, de Smet M, *et al.* The effects of a specific oral PAF-antagonist, MK-587, on antigen-induced early and late asthmatic reactions in man. *Am Rev Respir Dis* 1991; **143**: A811.

**Received 14 August 1992; accepted 14 September 1992**

## References

[PDF_00612] Abraham W. M., Stevenson J. S., Garrido R. (1989). A possible role for PAF in allergen-induced late responses: modification by a selective antagonist.. J Appl Physiol (1985).

[PDF_00615] Abraham W. M. (1988). The role of leukotrienes in allergen-induced late responses in allergic sheep.. Ann N Y Acad Sci.

[PDF_00557] Barnes P. J., Chung K. F., Page C. P. (1988). Platelet-activating factor as a mediator of allergic disease.. J Allergy Clin Immunol.

[PDF_00565] Björk J., Smedegård G. (1983). Acute microvascular effects of PAF-acether, as studied by intravital microscopy.. Eur J Pharmacol.

[PDF_00573] Chilton F. H., O'Flaherty J. T., Walsh C. E., Thomas M. J., Wykle R. L., DeChatelet L. R., Waite B. M. (1982). Platelet activating factor. Stimulation of the lipoxygenase pathway in polymorphonuclear leukocytes by 1-O-alkyl-2-O-acetyl-sn-glycero-3-phosphocholine.. J Biol Chem.

[PDF_00570] Cuss F. M., Dixon C. M., Barnes P. J. (1986). Effects of inhaled platelet activating factor on pulmonary function and bronchial responsiveness in man.. Lancet.

[PDF_00587] Gundel R. H., Kinkade P., Torcellini C. A., Clarke C. C., Watrous J., Desai S., Homon C. A., Farina P. R., Wegner C. D. (1991). Antigen-induced mediator release in primates.. Am Rev Respir Dis.

[PDF_00617] Gundel R. H., Torcellini C. A., Clarke C. C., Desai S., Lazer E. S., Wegner C. D. (1991). The effects of a 5-lipoxygenase inhibitor on antigen-induced mediator release, late-phase bronchoconstriction and cellular infiltrates in primates.. Adv Prostaglandin Thromboxane Leukot Res.

[PDF_00585] Gundel R. H., Wegner C. D., Letts L. G. (1992). Antigen-induced acute and late-phase responses in primates.. Am Rev Respir Dis.

[PDF_00627] Gundel R. H., Wegner C. D., Torcellini C. A., Letts L. G. (1992). The role of intercellular adhesion molecule-1 in chronic airway inflammation.. Clin Exp Allergy.

[PDF_00582] Heuer H. O., Casals-Stenzel J., Muacevic G., Weber K. H. (1990). Pharmacologic activity of bepafant (WEB 2170), a new and selective hetrazepinoic antagonist of platelet activating factor.. J Pharmacol Exp Ther.

[PDF_00609] Heuer H. O. (1991). Inhibition of active anaphylaxis in mice and guinea pigs by the new hetrazepinoic PAF antagonist bepafant (WEB 2170).. Eur J Pharmacol.

[PDF_00606] Lee T., Lenihan D. J., Malone B., Roddy L. L., Wasserman S. I. (1984). Increased biosynthesis of platelet-activating factor in activated human eosinophils.. J Biol Chem.

[PDF_00596] Lefer A. M., Roth D. M., Lefer D. J., Smith J. B. (1984). Potentiation of leukotriene formation in pulmonary and vascular tissue.. Naunyn Schmiedebergs Arch Pharmacol.

[PDF_00591] Naccache P. H., Molski M. M., Volpi M., Becker E. L., Sha'afi R. I. (1985). Unique inhibitory profile of platelet activating factor induced calcium mobilization, polyphosphoinositide turnover and granule enzyme secretion in rabbit neutrophils towards pertussis toxin and phorbol ester.. Biochem Biophys Res Commun.

[PDF_00559] Rubin A. H., Smith L. J., Patterson R. (1987). The bronchoconstrictor properties of platelet-activating factor in humans.. Am Rev Respir Dis.

[PDF_00624] Solèr M., Sielczak M. W., Abraham W. M. (1989). A PAF antagonist blocks antigen-induced airway hyperresponsiveness and inflammation in sheep.. J Appl Physiol (1985).

[PDF_00599] Voelkel N. F., Worthen S., Reeves J. T., Henson P. M., Murphy R. C. (1982). Nonimmunological production of leukotrienes induced by platelet-activating factor.. Science.

[PDF_00567] Wardlaw A. J., Moqbel R., Cromwell O., Kay A. B. (1986). Platelet-activating factor. A potent chemotactic and chemokinetic factor for human eosinophils.. J Clin Invest.

[PDF_00589] Wegner C. D., Jackson A. C., Berry J. D., Gillespie J. R. (1984). Dynamic respiratory mechanics in monkeys measured by forced oscillations.. Respir Physiol.

